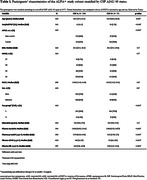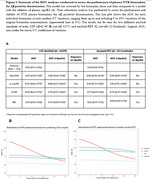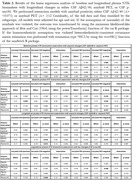# Performance of plasma NTK biomarkers to detect amyloid‐β pathology in cognitively unimpaired individuals at risk of Alzheimer’s disease

**DOI:** 10.1002/alz.090801

**Published:** 2025-01-09

**Authors:** Armand González Escalante, Marta Milà‐Alomà, Wagner Scheeren Brum, Nicholas J. Ashton, Paula Ortiz‐Romero, Carolina Minguillon, Mahnaz Shekari, Federica Anastasi, Marta del Campo, Clara Quijano‐Rubio, Gwendlyn Kollmorgen, Juan Domingo Gispert, Henrik Zetterberg, Natalia Vilor‐Tejedor, Kaj Blennow, Marc Suárez‐Calvet

**Affiliations:** ^1^ Barcelonaβeta Brain Research Center (BBRC), Pasqual Maragall Foundation, Barcelona Spain; ^2^ Universitat Pompeu Fabra, Barcelona Spain; ^3^ Hospital del Mar Research Institute (IMIM), Barcelona Spain; ^4^ Centro de Investigación Biomédica en Red de Fragilidad y Envejecimiento Saludable (CIBERFES), Madrid Spain; ^5^ Department of Psychiatry and Neurochemistry, Institute of Neuroscience and Physiology, The Sahlgrenska Academy, University of Gothenburg, Mölndal Sweden; ^6^ Graduate Program in Biological Sciences: Biochemistry, Universidade Federal do Rio Grande do Sul (UFRGS), Porto Alegre Brazil; ^7^ Department of Psychiatry and Neurochemistry, Institute of Neuroscience and Physiology, The Sahlgrenska Academy, University of Gothenburg, Mölndal, Gothenburg Sweden; ^8^ Centre for Age‐Related Medicine, Stavanger University Hospital, Stavanger Norway; ^9^ Institute of Psychiatry, Psychology and Neuroscience, Maurice Wohl Clinical Neuroscience Institute, King’s College London, London United Kingdom; ^10^ NIHR Biomedical Research Centre for Mental Health and Biomedical Research Unit for Dementia at South London and Maudsley, NHS Foundation, London United Kingdom; ^11^ Hospital del Mar Medical Research Institute (IMIM), Barcelona Spain; ^12^ Centro de Investigación Biomédica en Red de Fragilidad y Envejecimiento Saludable (CIBERFES), Instituto de Salud Carlos III, Madrid Spain; ^13^ Hospital del Mar Research Institute, Barcelona, Barcelona Spain; ^14^ Centre for Genomic Regulation (CRG), Barcelona Institute of Science and Technology (BIST), Barcelona Spain; ^15^ Departamento de Ciencias Farmacéuticas y de la Salud, Facultad de Farmacia, Universidad San Pablo‐CEU, CEU Universities, Madrid Spain; ^16^ Roche Diagnostics International Ltd., Rotkreuz Switzerland; ^17^ Roche Diagnostics GmbH, Penzberg Germany; ^18^ Centro de Investigación Biomédica en Red Bioingeniería, Biomateriales y Nanomedicina, Instituto de Salud Carlos III, Madrid Spain; ^19^ Centro Nacional de Investigaciones Cardiovasculares (CNIC), Madrid Spain; ^20^ Hong Kong Center for Neurodegenerative Diseases, Hong Kong China; ^21^ Department of Neurodegenerative Disease, UCL Queen Square Institute of Neurology, University College London, London, ‐ United Kingdom; ^22^ Wisconsin Alzheimer's Disease Research Center, University of Wisconsin School of Medicine and Public Health, Madison, WI USA; ^23^ UK Dementia Research Institute, University College London, London United Kingdom; ^24^ Department of Clinical Genetics, Erasmus University Medical Center, Rotterdam Netherlands; ^25^ Clinical Neurochemistry Laboratory Sahlgrenska University Hospital, Mölndal Sweden; ^26^ Servei de Neurologia, Hospital del Mar, Barcelona Spain; ^27^ Barcelonaβeta Brain Research Center (BBRC), Barcelona Spain

## Abstract

**Background:**

Alzheimer disease (AD) plasma biomarkers change in the preclinical stage of AD. However, the robustness of the discrimination performance of these biomarkers, as well as their association with longitudinal primary pathology (amyloid and tau) changes, is less understood. We aimed to determine the ability of baseline and longitudinal plasma amyloid‐β (Aβ)42/40, p‐tau181, GFAP and NfL to detect primary pathology in CU individuals at risk of AD.

**Method:**

Plasma biomarkers were measured using the NeuroToolKit (NTK), a panel of robust prototype biomarker assays (Roche Diagnostics International Ltd, Rotkreuz, Switzerland), in CU participants of ALFA+. We assessed their performance for detecting early signs of amyloid‐positivity (as defined by CSF Aβ42/40 <0.071 or amyloid PET ≥12 Centiloids) using ROC analyses. We simulated the impact of random variability on each biomarker’s detection performance. Finally, we tested the baseline and longitudinal association of plasma biomarkers with longitudinal changes of primary pathology over three years.

**Results:**

We included 403 CU participants (49.3‐73.6 years old), 135 (33.5%) were CSF amyloid‐positive (Table 1), and 345 had amyloid PET available (15.7% PET amyloid‐positive). Plasma Aβ42/40 had the highest performance for Aβ‐positivity discrimination [AUC(CSF):0.87, AUC(PET):0.90], followed by p‐tau181 [AUC(CSF):0.72, AUC(PET):0.81]. Adding plasma NTK ApoE4 significantly improved the performance of plasma p‐tau181 and GFAP [AUC: 0.79 and 0.81]. Plasma Aβ42/40 was the most sensitive biomarker to the addition of random variability, while the others remained moderately stable (Figure 1). In PET amyloid‐negative individuals, lower baseline plasma Aβ42/40 was associated with longitudinal increases in amyloid PET Centiloids and CSF p‐tau181. In the low‐Aβ‐burden group (CSF amyloid‐positive, PET amyloid‐negative), higher baseline plasma p‐tau181 was associated with longitudinal increases in CSF p‐tau181. Plasma GFAP changed concurrently with amyloid, both CSF and PET, before amyloid PET becomes positive (Table 2).

**Conclusions:**

Plasma Aβ42/40 had the best performance in detecting Aβ pathology, but its discrimination performance was more sensitive to measurement variability. Plasma Aβ42/40 and p‐tau181 predict longitudinal changes in amyloid and tau pathology. Plasma GFAP changed concurrently with amyloid pathology. Overall, our study showed that different blood‐based biomarkers offer distinct information in preclinical AD, making each of them valuable for diagnostic, prognostic or monitoring purposes.